# Evaluation of the Antimalarial Activity of the Hydroalcoholic Extract of Leaf of *Leonotis ocymifolia* (Burm. f.) Iwarsson (Lamiaceae) against *Plasmodium berghei* in Mice

**DOI:** 10.1155/2020/5384804

**Published:** 2020-09-17

**Authors:** Tewolde Teklu, Ephrem Engidawork, Teshome Nedi, Tilahun Teklehaymanot, Leake Gebremeskel

**Affiliations:** ^1^Department of Pharmacy, College of Health Sciences, Aksum University, Aksum, Ethiopia; ^2^Department of Pharmacology and Clinical Pharmacy, School of Pharmacy, College of Health Sciences, Addis Ababa University, Addis Ababa, Ethiopia; ^3^Aklilu Lemma Institute of Pathology, Addis Ababa University, Addis Ababa, Ethiopia

## Abstract

Malaria's global impact, fueled by resistance to several antimalarial drugs, has necessitated a quest to new antimalarial drugs from several sources with traditional medicinal plants being one of them. This study was conducted to assess the antimalarial activity of a traditionally used medicinal plant, *Leonotis ocymifolia,* against *Plasmodium berghei*. The plant has been extracted using maceration technique, and doses ranging from 100–800 mg/kg of *Leonotis ocymifolia* were used to test its antimalarial activity. Tween 80 (2% in water) and chloroquine 25 mg/kg were used as negative and positive controls, respectively. The antimalarial activities of the plant were determined by measuring parasitemia, survival time, packed cell volume, temperature, and weight. The plant's hydroalcoholic extract, as compared to negative control, maximally decreased parasite load by 41.4% at 800 mg/kg (*p* < 0.001). This parasite suppression was followed by longer survival time in the groups taking 400 mg/kg (*p* < 0.05) and 800 mg/kg (*p* < 0.05) in a four-day suppressive test and in those taking 800 mg/kg (*p* < 0.05) in Rane's test. The plant did not prevent weight and PCV reduction but prevented temperature reduction at 400 mg/kg (*p* < 0.05) and 800 mg/kg (*p* < 0.05) in a four-day suppressive model, and at 800 mg/kg (*p* < 0.05) in Rane's model. The average but consistent antimalarial activity of the plant across the test models corroborates the folkloric antimalarial use of the plant. The study recommends further pharmacological screenings, isolation, and identification of active compound(s) of the plant *Leonotis ocymifolia*.

## 1. Introduction

Malaria is one of the major global health problems, and despite headways, it still remains one of the deadly diseases [1]. Due to resistance of malaria parasites against the well-known available drugs, the chemotherapy of malaria has become more complex and challenging [2]. In addition to the clinically approved drugs, different plants are traditional used to prevent, cure, and/or alleviate symptoms of malaria. Among these plants is *Leonotis ocymifolia*, locally called “Raskmir,” which has several ethnobotanical uses, including malaria [3], abdominal pain [4] headache [5], hypertension and asthma [6], diabetes, eczema, and purgative, [7], anthrax and wound [8], and diarrhea [9]. Moreover, various pharmacological investigations have also indicated the plant to have antiplasmodial [10], trypanocidal, antifertility and anti-implantation activities [11]. Although *in vitro* studies have indicated the plant to have a potential antiplasmodial effect [10, 12], it has not been as yet investigated *in vivo.* Hence, in this study, the antimalarial effect of the hydroalcoholic extract of the plant was investigated in an effort to contribute to the discovery of new drugs with high activity and low toxicity.

## 2. Materials and Methods

### 2.1. Plant Material Collection and Authentication

The plant's leaf was collected from Mount Entoto, Addis Ababa. Identification and authentication of the collected plant was conducted at the National Herbarium, College of Natural Sciences and Computation, Addis Ababa University, and the voucher specimen was deposited with the voucher number TT003.

### 2.2. Preparation of Extract

The fresh leaf of the plant was cleaned, dried in shaded area, and was then ground into a coarse powder using mortar and pestle. Two hundred grams of the course plant material was weighed by sensitive digital weighing balance (Mettler Toledo, Switzerland) and extracted by cold maceration technique. The plant material was soaked in a separate flask containing 80% methanol (1 : 5 (*w*/*v*)) and then was placed on a shaker (Bibby Scientific Limited, Stone, Staffordshire, UK) tuned to 120 rpm for 72 h at room temperature. The extract was filtered using Whatman grade No. 1 filter paper (Schleicher and Schuell MicroScience GmbH, Germany), and marcs were re-extracted for a second and third time by adding another fresh methanol in water. The filtrates were combined and concentrated in a rotary evaporator (Buchi Rotavapor R-200, Switzerland) with temperature not exceeding 40°C. Finally, the extract was transferred into vials and kept in a desiccator until use.

### 2.3. Experimental Animals

A total of 128 mice (male) for the antimalarial test and 5 females (for acute toxicity) were used in the present study. Swiss albino mice aged 6 to 8 weeks obtained from the animal house of the Department of Pharmacology and Clinical Pharmacy, School of Pharmacy, Addis Ababa University, were used. The animals were kept at room temperature with 12 h light/dark cycle, with food and water *ad libitum*. They were acclimatized for seven days in the laboratory before being used for the experiments. The care and handling was according to international guidelines for the use and maintenance of experimental animals [13–15]. Ethical approval was obtained from health research and ethics review committee of the College of Health Sciences, Addis Ababa University, Ethiopia.

### 2.4. Acute Oral Toxicity Test

An acute oral toxicity test was conducted according to the procedure of the OECD guideline 425 [15]. Five female albino mice were used for the test. All animals were fasted for 3 h before and 1 h after the administration of the extract. First, one animal was administered 2 gm/kg. Since no death was observed within 24 h, additional four animals were added and administered the same dose. The animals were observed continuously for 4 h with a 30 min interval whether they show hair erection, tremors, convulsions, salivation, lacrimation, diarrhea, lethargy, and reduction in feeding as well as mortality and then followed for 14 consecutive days with an interval of 24 h.

### 2.5. Grouping and Dosing

In the four-day suppressive test, 48 mice were randomly divided into six groups of eight mice per group and were categorized as follows: the first group (Group I) was designated as negative control and administered 10 mL/kg of 2% Tween 80 in water. The second, third, fourth, and fifth groups (Group II–V) were orally treated with 100 mg/kg, 200 mg/kg, 400 mg/kg, and 800 mg/kg, respectively. One group (Group VI) was assigned as positive control and treated with chloroquine 25 mg/kg. The same grouping was used in Rane's test and the prophylactic test except that the lowest dose (100 mg/kg) was dropped after the four-day suppressive test as it was not having significant effect. In all models, the extract was dissolved in a mixture of distilled water and Tween 80. After fully dissolving it, it was given to the animals based on their weight. The treatment, which was carried out once every 24 hours (at the same time in each day), was conducted by oral means of administration using oral gavage.

### 2.6. Four-Day Suppressive Test

The Peters four-day suppressive test against mice infected with chloroquine sensitive *Plasmodium berghei* (chloroquine sensitive *P*. *berghei* ANKA obtained from the Ethiopian Public Health Institute (EPHI) was employed according to the method described by Peters et al. [13]. Forty-eight mice were intraperitoneally injected with inoculum of 1 × 10^7^*P*. *berghei-*infected erythrocytes on the first day (Day 0). After Two-hour postinfection, the mice were randomly distributed into six groups and treated as described in grouping and dosing section. Treatment was continued for additional three consecutive days (until Day 3). On Day 4 of the experiment, blood was collected from the tail of each mouse and smear was prepared on a microscope slide to determine parasitemia. In addition, weight, temperature, and packed cell volume (PCV) were measured just before infection and at the end of the experiment. Afterwards, mice were followed for 28 days (Day 0–Day 27) so that the mean survival time for each group could be determined.

### 2.7. Rane's Test

Rane's test, which evaluates the curative potential of extracts, was carried out according to the method described by Ryley and Peters [16]. For each plant extract, 40 male mice were injected intraperitoneally with inoculum of 1 × 10^7^*P*. *berghei-*infected erythrocytes on the first day (Day 0). After 72 h (Day 3), the animals were randomly assigned into five groups with eight mice in each group and treated for four days with their respective doses as described in grouping and dosing section. Weight, temperature, and PCV were measured just before the first dose and at the end of the experiment (Day 7), and parasitemia was measured daily throughout the experiment (beginning from Day 3). Thereafter, all groups were followed for 28 days and survival time was recorded.

### 2.8. Prophylactic Test

Evaluation of the prophylactic potential of the extract was carried out according to the method described by Peters [17]. For each plant extract, 40 male mice were randomly distributed into five groups of eight mice each and treated as described earlier undergrouping and dosing section. The test animals received treatment for three days prior to infection. Following the completion of treatment, the animals were inoculated with *P*. *berghei-*infected erythrocytes (1 × 10^7^), and this day was assigned as Day Zero (D0). The animals were kept in the animal house for three days (D0, D1, and D2). The next day (Day 3), blood smears were prepared from each mouse and parasitemia level was determined. Weight, temperature, and PCV were recorded on Day 0 and Day 3. Finally, the groups were followed for 28 days in order to record their survival time.

### 2.9. Parasitemia and Survival Time Measurement

Blood was collected from the tail snip of each mouse, and thin smear was prepared on microscope slides. After air drying, the smears were fixed with absolute methanol and stained with 10% Giemsa stain for 15 min. The slides were then gently rinsed with tap water and dried at room temperature. With little drop of oil immersion, the number of pRBC was counted using light microscope (Olympus N-120A, Philippines) with an objective lens of 100x magnification power. Five fields of approximately 100–200 cells in each slide were counted [18, 19], and taking the average count, percent parasitemia was calculated using the formula indicated below as described elsewhere [20]:

(1) $$\begin{array}{c} \%\text{ parasitemia}=\frac{\text{number of parasitized RBC }}{\text{total number of RBC count}}\times 100. \end{array}$$

Afterwards, percent parasitemia suppression of the extract as compared to negative controls was calculated using the following formula [20]:

(2) $$\begin{array}{c} \%\text{ suppression }=\frac{\text{\% parasitemia in NG}-\text{\% parasitemia in SG}}{\text{\% parasitemia in negative control}}\;\times 100, \end{array}$$

where NG is the negative control and SG is the study group.

Finally, the animals were followed and their mean survival times (MST) were measured using the formula indicated below as described elsewhere [21]:

(3) $$\begin{array}{c} \text{MST}=\frac{\text{sum of survival time of all mice in a group}}{\text{total number of mice in that group}}. \end{array}$$

### 2.10. Data Analysis

Results are expressed as mean ± standard error of the mean (SEM). The experimental results were analyzed using the software Statistical Package for Social Sciences (SPSS), version 20. Statistical significance was determined by one-way analysis of variance (ANOVA) followed by the Tukey post hoc test to compare the levels of parasitemia, survival time, changes in body weight, PCV, and rectal temperature. *p* value of less than 0.05 was considered statistically significant. The analyzed data were then presented using tables and figures.

## 3. Results

### 3.1. Acute Oral Toxicity Test

The hydroalcoholic leaf extract of *Leonotis ocymifolia* caused no gross behavioral changes and mortality within 24 h as well as in the next 14 days, indicating that the LD50 of the extract was greater than 2000 mg/kg.

### 3.2. Effect in the Four-Day Suppressive Test

With the exception of the group taking the smallest dose (100 mg/kg), all other groups were having significantly lower parasitemia than the negative control (*p* < 0.001). Comparison among the degree of suppression among the extract's doses showed that there was higher degree of suppression by 400 mg/kg against the degree of suppression by 100 mg/kg (<0.01) and 200 mg/kg (*p* < 0.01). Similarly, the largest dose (800 mg/kg) caused significant suppression than 100 mg/kg (*p* < 0.001) and 200 mg/kg (*p* < 0.001) (Table 1).

Analysis of survival time indicated that groups which took the higher two doses (400 mg/kg and 800 mg/kg) significantly outlived (*p* < 0.05) their negative control counterpart. Moreover, comparison among the extract's doses indicated that 400 mg/kg and 800 mg/kg caused a significantly longer survival time (*p* < 0.05) as compared to 100 mg/kg. The positive control was also having significant effect both against the negative control (*p* < 0.001) and the extract's dose (*p* < 0.001) Table 1.

**Figure 1:**
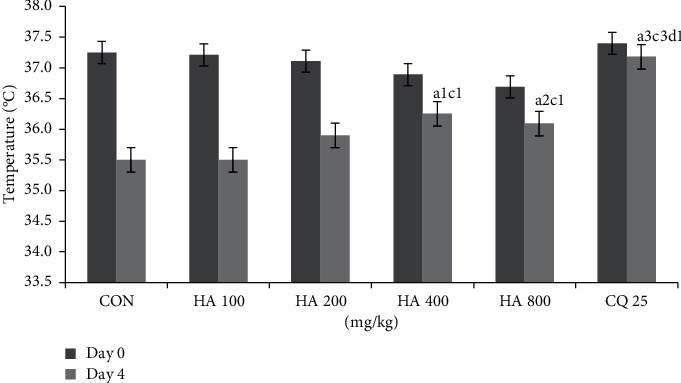
Rectal temperature of *P. berghei*-infected Swiss albino mice treated with hydroalcoholic extract of leaf of *Leonotis ocymifolia* in the four-day suppressive test; ^a^compared to negative control; ^c^compared to 100 mg/kg; ^d^compared to 200 mg/kg; ^1^*p* < 0.05, ^2^*p* < 0.01, and ^3^*p* < 0.001; 0 C: degree centigrade; HA: hydroalcoholic; CQ: chloroquine.

Measurement of body weight at the end of the test (D4) showed that all animals in all groups were found to have lower body weight than their initial weight (DO). Analysis of this change in body weight indicated that none of the doses of the extract were able to prevent weight reduction. Likewise, despite the lower loss of PCV compared to the negative control, none of extract-treated groups had better PCV. With regards to temperature change, the larger doses (400 mg/kg and 800 mg/kg) were able to significantly prevent reduction of temperature as compared to the negative control as well as the smallest dose (100 mg/kg). The positive control, however, was significantly effective at preventing the loss of weight (*p* < 0.01), PCV (*p* < 0.01) (Table 2), and temperature (*p* < 0.001) as compared to the negative control (Figure 1).

### 3.3. Effect in Rane's Test

**Figure 2:**
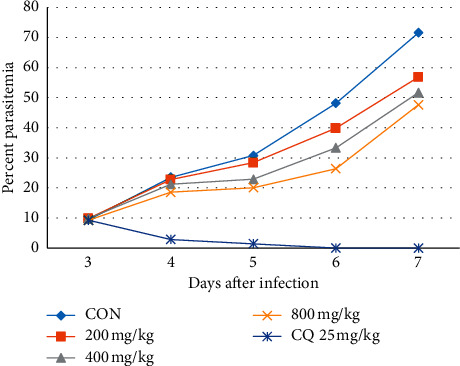
The effect of hydroalcoholic leaf extract of *Leonotis ocymifolia* on parasitemia in Rane's test. CON, negative control; CQ, chloroquine.

Both the extract-treated groups and negative control showed a continued rise in the load of parasitemia (Figure 2). However, there was a difference in the level of mean parasitemia among them, and analysis of this difference indicated that parasitemia was significantly reduced with all doses of the extract (*p* < 0.001) as well as the standard (*p* < 0.001) compared to the level in the negative control. There was also significantly lower parasitemia in the groups taking the largest dose (800 mg/kg; *p* < 0.05) and positive control (*p* < 0.001) than the group that took 200 mg/kg. Concerning survival time, analysis of the survival time data pointed out that only the highest dose (*p* < 0.05) and the standard (*p* < 0.001) were able to significantly prolong survival time as compared to the negative control (Table 3).

**Figure 3:**
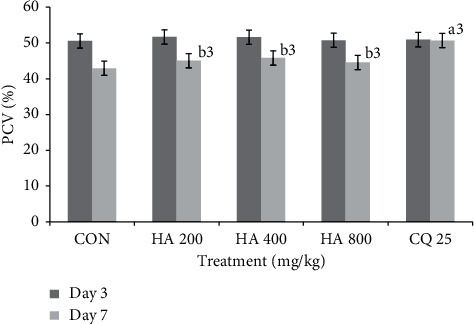
Comparison of packed cell volume of *P. berghei*-infected Swiss albino group treated with hydroalcoholic extract of leaf of *Leonotis ocymifolia* in Rane's test.

There was a consistent daily decrease in weight across all doses and negative control. Comparison of the degree of decrease in weight among both the doses and the negative control indicated that none of the doses of the extract were able to significantly prevent weight reduction. Similarly, unlike the standard drug, the extract did not prevent PCV reduction significantly compared to negative control (Figure 3). Pertaining to temperature change, the largest dose (800 mg/kg, *p* < 0.05) prevented the reduction in body temperature as compared to negative control (Table 4).

### 3.4. Effect in the Prophylactic Test

In comparison to the negative control, there was significantly lower parasitemia in all extract-treated groups (*p* < 0.001) (Table 5). Comparison of the level of suppression among the extract's doses indicated that both 400 mg/kg and 800 mg/kg were having significant suppression (*p* < 0.001) compared to 200 mg/kg. In Vis-à-vis survival time, the extract was not able to significantly extend the survival time of the rodents beyond that of the negative control. The positive control, however, had a positive impact at extending the survival time of the rodents (*p* < 0.05) (Table 5).

In the prophylactic test, unlike in the other two tests, comparison between the extract and control revealed that the extract, at all doses, was not able to prevent reduction of all parameters (weight, temperature, and PCV). Multiple comparison among the doses indicated that there was no significant difference in terms of preventing reduction in temperature, weight, and PCV. The positive control was able to sustain the initial measurement of all the three parameters as compared to negative control and 200 mg/kg (Table 6).

## 4. Discussion

The toxicity of several medicinal plants have been well documented [22, 23]. Hence, the safety status of all potential medicinal plants has to be investigated before further exploration. Based on the OECD guideline 425 [15] of acute toxicity studies, the LD50 was found to be > 2000 mg/kg. Since the LD50 of the plant's hydroalcoholic extract was found to be more than three times the minimum effective dose (200 mg/kg), it was taken as a good candidate for further studies [24, 25].

Three models have been used to test the antimalarial effect of the plant, one model being the four-day suppressive test. This test evaluates the potential schizontocidal activity of test compounds [13], and using this model, the hydroalcoholic extract of *Leonotis ocymifolia* had a maximum suppression of 41.4% at 800 mg/kg. Compounds that reduce parasitemia load by 30% or more are considered as having schizontocidal activity against malaria [18]. By this standard, the hydroalcoholic extract of the plant can be considered to be active in its schizontocidal activity against malaria. In this test, maximum parasite suppression was achieved by positive control (chloroquine). The significantly lower parasitemia suppression by the extract compared to the positive control could be due to low level of active compound(s) associated with the crude nature of the extract [18]. The higher doses of the extract, compared to the negative control, had resulted in significantly higher survival time. This relationship of dose-versus-survival time may be related with the higher degree parasite suppression which, hence, resulted in mitigation of its deleterious pathologic consequences [19].

The potential of the plant to prevent further malaria attacks in an established infection was investigated using another model known as Rane's test [16]. In this test, although there was no cure, there was significant reduction in parasitemia with all the doses used. The level of parasitemias in the extract-treated groups was consistently lower than those in the negative control. While this may indicate that the plant had rapid onset of action [26], the overall lower curative than suppressive effect could possibly be due to too short duration of action to contain the exponentially growing parasites in an established infection [19]. The survival time was significantly prolonged only at the highest dose which could probably be related to the relatively higher parasitemia reduction by this dose.

The third model, the prophylactic test, was conducted to see if the extract can prevent parasitemia proliferation. In this test, the extract was able to hinder the growth of parasitemia at all the doses used. The degree of the suppression was, however, smaller than seen in the two previous models. This effect may have been related with the fact that the extract is administered prior to infection establishment and hence may have been rapidly metabolized and/or excreted [27–29]. Similar results, in which plants had better suppressive effect than prophylactic effect were reported in other studies [30, 31]. The extract, in spite of the parasitemia suppression, was not able to prolong the survival time compared to controls which points out that the degree of suppression was not large enough to maintain the overall well-being of the rodents.

If intervention is withheld, malaria typically reduces body weight [19], temperature [19, 32], and PCV [33]. An effective medicinal extract is, therefore, expected to lessen or prevent these reductions. This study has indicated that the hydroalcoholic extract of the plant was not able to prevent the drop of PCV. The failure of the extract to prevent PCV reduction could probably be associated with the hemolytic effect of saponin contained in the extract [34, 35].

Weight decrement is another typical symptom of malaria and has been associated with decreased food intake, disturbed metabolic function, and hypoglycemia [19, 36]. Weight of the rodents was being weight in all models. The extract was able to decrease, albeit not significant, the level of parasitemia compared to negative control. This failure to prevent weight reduction may be related with saponins and tannins that the plant contains saponins are known to hinder protein utilization by inhibiting digestive enzymes such as trypsin and/or by forming saponin-protein complexes Similarly, tannins, through formation of protein-tannin complexes, may have diminished nutrient utilization and reduced food intake [35]. This is similar with the findings of other plants such as *Dodonaea angustifolia* seed extract [20]and *Calpurnia aurea* leaf extract [37]where these plants had significant parasitemia suppression but were unable to prevent the drop of weight.

Without intervention, there is, unlike in humans, a continual reduction in temperature due to malaria in rodents [32]. The plant, at higher doses, prevented temperature reduction in both suppressive and Rane's test models. It did not, however, have an effect during the preventive model which might be associated with the lower parasitemia suppression seen in this model. Similar study results, in which plants have similar degree of suppression but failed to prevent temperature reduction, were reported in other studies [34, 37].

Bioactive constituents are often attributed to the therapeutic effect of medicine plants [38]. Plants known to have antimalarial effect have been explored, and many secondary metabolites have been identified [39, 40]. In a previous study, the hydroalcoholic extract of the plant was screened for its phytochemical content and shown to have terpenoids, steroids, alkaloids, tannins, flavonoids, and polyphenol compounds [41]. Several studies have indicated that these metabolites have antiplasmodial activities. Considering these reports and the antiplasmodial activities of the plant seen in this study, the antiplasmodial effect of *Leonotis ocymifolia* may be attributed to its secondary metabolites.

## 5. Conclusion

According to the results, the plant is safe in rodents and possesses antimalarial activity. The safety and antimalarial activities prove the traditional use and *in vitro* antiplasmodial activity. The plant has promising antimalarial activity; therefore, it is recommended that further studies be done to isolate and identify pharmacologically active principle(s) responsible for the antimalarial activities.

## Figures and Tables

**Table 1:** Antimalarial activity test of the hydroalcoholic extracts of leaf of *Leonotis ocymifolia* against *P*. *berghei* in the four-day suppressive test.

Treatment (mg/kg)	Parasitemia (%)	Suppression (%)	MST±SEM (days)
CON	38.79±4.1	—	6.88±1.55
HA 100 mg/kg	37.49±2.81	6.5^*d*3^	6.75±1.49
HA 200 mg/kg	29.56±3.06	23.33^*a*3^	7.25±1.39
HA 400 mg/kg	24.46±3.30	36.55^*a*3*c*3*d*2^	8.88±1.55^*a*1*c*1^
HA 800 mg/kg	22.59±3.21	41.4^*a*3*c*3*d*3^	8.75±0.31^*a*1*c*1^
CQ 25 mg/kg	000±0.00	100^*a*3*c*3*d*3*e*3*f*3^	28.00 ± 0.00^*a*3*c*3*d*3*e*3*f*3^

Data are expressed as mean ± SEM (*n* = 8); ^*a*^compared to negative control; ^*b*^compared to CQ 25 mg/kg; ^*c*^compared to 100 mg/skg; ^*d*^compared to 200 mg/kg; ^*e*^compared to 400 mg/kg; ^*f*^compared to 800 mg/kg: ^1^*p* < 0.05, ^2^*p* < 0.01, and ^3^*p* < 0.001; HA, hydroalcoholic; negative controls received 2% Tween 80 in water; CON, negative control; CQ, chloroquine.

**Table 2:** Body weight, rectal temperature, and packed cell volume of *P*. *berghei*-infected Swiss albino mice treated with hydroalcoholic extract of leaf of *Leonotis ocymifolia* in the four-day suppressive test.

Treatment	Weight			Packed cell volume (PCV)		
	D0	D4	Change (%)	D0	D4	Change (%)
CON	31.52±1.91	29.01±1.70	−7.72	53.48±3.01	49.24±3.10	−8.11
HA 100 mg/kg	34.06±1.14	31.40±0.78	−7.59	52.13±0.87	47.98±0.99	−8.33
HA 200 mg/kg	28.99±1.04	27.10±0.99	−6.51	54.28±1.51	50.31±1.89	−7.21
HA 400 mg/kg	33.55±1.06	32.46±0.90	−3.11	52.31±1.47	49.90±1.14	−4.43
HA 800 mg/kg	33.05±1.82	31.72±1.69	−3.97	54.40±1.47	51.55±1.68	−5.28
CQ 25 mg/kg	31.52±0.61	31.57±1.46	0.31^*a*2*c*2*d*2^	54.16±1.31	53.84±1.16	−0.51^*a*1*c*1*d*1^

Data are expressed as mean ± SEM (*n* = 8); ^*a*^compared to negative control; ^*b*^compared to CQ 25 mg/kg; ^*c*^compared to 100 mg/kg; ^*d*^compared to 200 mg/kg; ^*e*^compared to 400 mg/kg; ^*f*^compared to 800 mg/kg: ^1^*p* < 0.05, ^2^*p* < 0.01, and ^3^*p* < 0.001; HA, hydroalcoholic; negative controls received 2% Tween 80 in water; CON, negative control; CQ, chloroquine.

**Table 3:** Antimalarial activity test of the hydroalcoholic extracts of leaf of *Leonotis ocymifolia* against *P*. *berghei* in Rane's test.

Treatment dose (mg/kg)	Suppression (%)	MST±SEM (days)
CON	—	7.25±0.46
HA 200 mg/kg	20.57^*a*3^	7.5±0.92
HA 400 mg/kg	27.75^*a*3^	8.25±1.39
HA 800 mg/kg	33.44^*a*3*d*2^	8.63±0.74^*a*1^
CQ 25 mg/kg	97.10^*a*3*d*3*e*3*f*3^	28±00^*a*3*d*3*e*3*f*3^

Data are expressed as mean ± SEM (*n* = 8); ^*a*^compared to negative control; ^*b*^compared to CQ 25 mg/kg; ^*d*^compared to 200 mg/kg; ^*e*^compared to 400 mg/kg; ^*f*^compared to 800 mg/kg: ^1^*p* < 0.05, ^2^*p* < 0.01, and ^3^*p* < 0.001; HA, hydroalcoholic; CON, negative control; CQ, chloroquine; MST, mean survival time.

**Table 4:** Body weight and rectal temperature of *P*. *berghei*-infected Swiss albino group treated with hydroalcoholic extract of leaf of *Leonotis ocymifolia* in Rane's test.

Treatment (mg/kg)	Body weight			Temperature		
	Day 3	Day 7	Change (%)	Day 3	Day 7	Change (%)
CON	35.32±0.96	32.37±0.99	−8.39	37.15±0.09	32.88±0.66	−8.3
HA 200 mg/kg	33.19±1.14	31.49±1.73	−5.20	37.09±0.12	33.66±0.37	−6.1
HA 400 mg/kg	32.75±1.35	31.64±1.20	−3.26	37.14±0.12	34.77±0.31	−3.3
HA 800 mg/kg	30.74±0.68	29.33±0.74	−4.64	37.03±0.21	35.03±0.26	−3.1^*a*1^
CQ25 mg/kg	32.01±0.88	32.26±0.76	0.91^*a*1^	37.04±0.12	36.82±0.29	3.1^*a*3*d*3*e*2*f*1^

Data are expressed as mean ± SEM (*n* = 8); ^*a*^compared to negative control; ^*b*^compared to CQ 25 mg/kg; ^*d*^compared to 200 mg/kg; ^*e*^compared to 400 mg/kg; ^*f*^compared to 800 mg/kg: ^1^*p* < 0.05, ^2^*p* < 0.01, and ^3^*p* < 0.001; HA, hydroalcoholic; CON, negative control; CQ, chloroquine.

**Table 5:** Antimalarial activity test of the hydroalcoholic extracts of leaf of *Leonotis ocymifolia* against *P*. *berghei* in prophylactic test.

Treatment (mg/kg)	Parasitemia (%)	Suppression (%)	MST±SEM (days)
CON	20.77±3.12	—	6.62±1.06
HA 200 mg/kg	17.44±0.57	16.01^*a*3^	7.25±1.39
HA 400 mg/kg	13.84±0.55	25.76^*a*3*d*3^	7.38±1.06
HA 800 mg/kg	13.17±0.35	29.36^*a*3*d*3*e*1^	7.25±1.28
CQ25 mg/kg	0.11±0.15	99.40^*a*3*d*3*e*3*f*3^	8.70±0.64^*a*1^

Data are expressed as mean SEM (*n* = 8); analysis was performed with one-way ANOVA followed by the Tukey test; ^*a*^compared to negative control; ^*b*^compared to CQ 25 mg/kg; ^*d*^compared to 200 mg/kg; ^*e*^compared to 400 mg/kg; ^*f*^compared to 800 mg/kg: ^1^*p* < 0.05, ^2^*p* < 0.01, and ^3^*p* < 0.001; HA, hydroalcoholic; negative controls received 2% Tween 80 in water; CON, negative control; CQ, chloroquine.

**Table 6:** Body weight, rectal temperature, and packed cell volume of *P*. *berghei-infected* Swiss albino group treated with hydroalcoholic extract of leaf of *Leonotis ocymifolia* in prophylactic test.

Treatment (mg/kg)	Body weight			Temperature			Packed cell volume (PCV)		
	Day 0	Day 3	Change (%)	Day 0	Day 3	Change (%)	Day 0	Day 3	Change (%)
CON	36.98±0.45	35.1±0.44	−5.14	36.98±0.45	35.06±0.46	−5.24	54.94±3.84	49.70±4.20	−5.24
HA 200 mg/kg	37.03±0.5	35.54±0.47	−4.05	37.03±0.5	35.48±0.57	−4.18	56.29±4.72	53.13±6.60	−3.15
HA 400 mg/kg	36.68±1.16	35.69±1.11	−2.71	36.76±1.13	35.51±1.18	−3.34	53.96±1.83	52.53±1.54	−1.44
HA 800 mg/kg	37.34±0.51	36.35±1.59	−2.65	37.31±0.55	36.11±1.78	−3.24	54.55±1.36	52.12±1.34	−2.43
CQ 25 mg/kg	36.86±0.61	36.74±0.71	0.34^*a*3*d*2^	36.86±0.61	36.74±0.713	−0.33^*a*2*d*1^	52.45±2.79	52.20±3.19	−0.25^*a*2*d*2^

Data are expressed as mean ± SEM (*n* = 8); ^*a*^compared to negative control; ^*b*^compared to CQ 25 mg/kg: ^1^*p* < 0.05, ^2^*p* < 0.01, and ^3^*p* < 0.001; HA, hydroalcoholic; CON, negative control; CQ, chloroquine.

## Data Availability

The data used to support the findings of this study are available from the corresponding author upon request.

## Abbreviations

ANOVA: Analysis of variance
OECD: Organization for Economic Cooperation and Development
SEM: Standard error of the mean.
PCV: Packed cell volume
MST: Mean survival time
HA: Hydroalcoholic
CON: Negative control
CQ: Chloroquine.

## Supplementary Materials

Supplementary Materials Figure 1: *Leonotis ocymifolia* (Burm. f.) Iwarsson (Lamiaceae) in its natural habitat. Figure 2: parasite viewed at microscope which depicts the presence of parasitemia on Day 0 of mice taking 100 mg/kg in the four-day suppression test.
