# Supplementary material for: Evaluation of the Antimalarial Activity of the Hydroalcoholic Extract of Leaf of Leonotis ocymifolia (Burm. f.) Iwarsson (Lamiaceae) against Plasmodium berghei in Mice
Source: Evid Based Complement Alternat Med. 2020 Sep 17;2020:5384804. doi: 10.1155/2020/5384804 (PMC7519447; doi:10.1155/2020/5384804)
Supplement: Supplementary Materials — Figure 1: Leonotis ocymifolia (Burm. f.) Iwarsson (Lamiaceae) in its natural habitat. Figure 2: parasite viewed at microscope which depicts the presence of parasitemia on Day 0 of mice taking 100 mg/kg in the four-day suppression test. [file 5384804.f1.zip › Materials/Supplematary 1 photograph of the plant and paracetemia at four day suppression test.docx]

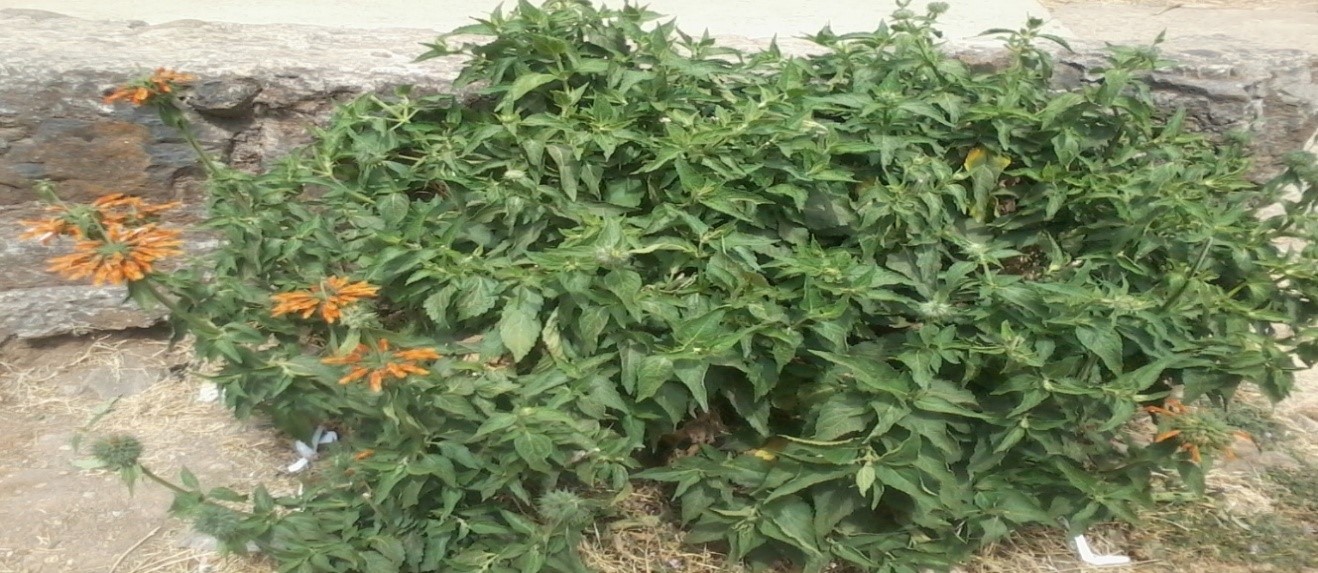


**Figure1**  : **Leonotis ocymifolia (Burm.f.) Iwarsson (Lamiaceae) in its natural habitat.**

It is a shrub 1-5 m high from a swollen woody base with leaves petiolate and orange flowers crowded spherical many-flowered verticils. The plant is found widespread from East Africa to South Africa.


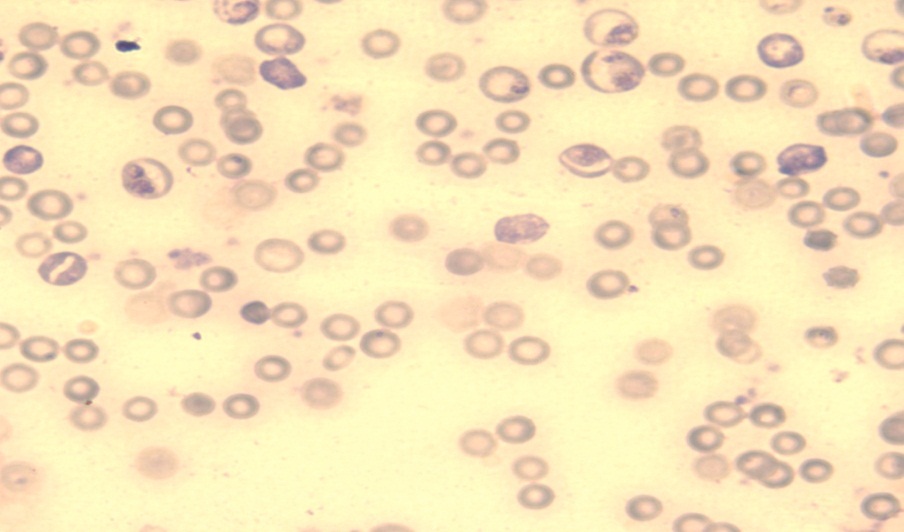


**Figure 2: Parasite viewed at Microscope** **which depicts the presence of parasitemia on day 0 of a mice taking 100mg/kg in four day suppression test**
